# Understanding response of microbial communities to saltwater intrusion through microcosms

**DOI:** 10.1016/j.csbj.2021.01.021

**Published:** 2021-01-21

**Authors:** Dandan Izabel-Shen

**Affiliations:** Department of Ecology, Environment and Plant Sciences, Stockholm University, Stockholm, Sweden

**Keywords:** Bacteria, Community assembly, Functional redundancy, Dispersal, Nitrogen genes

## Abstract

A central pursuit of microbial ecology is to accurately describe and explain the shifts in microbial community composition and function that occur in response to environmental changes. This goal requires a thorough understanding of the individual responses of different species and of the processes guiding the assembly of microbial populations similar in their response traits and corresponding functional traits. These research topics are addressed and synthesized in this Highlights, in four studies applying a trait-based framework to assess how environmental change affected the composition and functional performance of bacterioplankton of natural origin in microcosm experiments. The salinity of many aquatic environments is currently changing, due to climate change and anthropogenic activities. The mechanisms by which salinity influences community assembly, functional redundancy and functional genes involved in nitrogen cycle, and how dispersal modifies community outcome are explored in the four studies. Together, the findings of these case studies demonstrate the feasibility of using novel experiments in combination with integrative analyses of 16S rRNA and meta-‘omic’ data to address ecological questions. This combined approach has the potential to elucidate both the processes contributing to bacterial community assembly and the possible links between the compositional and functional changes that occur under shifting environmental conditions.

Microbes are the basis of every ecosystem on Earth and the global engines of biochemical cycles [1]. They are often taxonomically diverse, with their community composition determined by the complex interplay between environmental factors and ecological interactions. Microbial community composition varies over space and time [2]. The assembly processes driving this variability are unclear, as the multiple mechanisms complicate the identification of causal relationships [3], [4]. Moreover, the relationships between biodiversity and ecological functioning depend on environmental context. Shifts in microbial composition impacted the ecosystem processes in some systems but not in others [5]. Because fundamental roles of microbes in driving ecosystem processes, unraveling the mechanisms that underlie changes in microbial community composition under shifting environmental conditions can provide insights into the potential for ecosystem to recover. A better understanding of when and where the communities are functionally relevant is critical in guiding efforts aimed at maintaining microbial diversity and ecosystem multifunctionality.

Aquatic environments are typically heterogenous, characterized by nutrient and physio-chemical gradients of varying strength. These result in multiple niches harboring microorganisms that differ in their ecological strategies and functional traits. In this Highlights, I synthesized the methods and major findings of the four studies that explored the community assembly processes, dynamics and functional consequences of aquatic bacterioplankton subjected to changes in the surrounding environment in laboratory setting [6], [7], [8], [9]. The four published studies were chosen because of similarity in experimental setting. First, the aquatic environment allows the use of dialysis bags (i.e., studies I-III). Second, salinity is recognized as a key abiotic factor determining microbial composition [10], particularly along estuarine gradients, e.g., Baltic Sea, St. Lawrence Estuary and Minjiang River. Therefore, I predicted that changes in salinity would quickly select for distinct microbial communities. Third, the main goal of those studies was to examine the responses of bacterial communities to changes in salinity that occur in natural environments. This assessment provides insights into the ecological consequence of microbes after saltwater intrusion and the potential for ecosystem management. Last, the four studies applied trait-based approaches to understand community outcomes after a disturbance. To conclude, I will advocate a new perspective of this synthesis by highlighting the importance of trait-based approaches to elucidate the link between microbial composition and function under shifting environmental conditions.

Many coastal habitats are threatened by salinization due to climate change and/or anthropogenic activity [11]. Understanding the mechanisms by which salinity influences microbial communities and how compositional response is translated into ecosystem functioning, are necessary to move toward the goal of microbial community management. Three scenarios of saltwater intrusion were broached in the four studies to understand the ecological consequences of aquatic bacterioplankton. Scenario 1 illustrates that communities merely experience the surrounding environmental change, where no dispersal of cells between communities occurs in the recipient environment (Fig. 1A). Hence, study I & II determined the fitness and functional changes of locally adapted bacterial communities in the face of new environments. Scenario 2 exemplifies that communities experience coalescence, where not only the mixing of cells between communities but also respective environmental matrices occur (Fig. 1B). Study III examined whether and how the character and strength of dispersal impacted compositional responses to environmental change. Lastly, scenario 3 demonstrates an event of press disturbance, where a community experience varying levels of salt addition over a long period of time (Fig. 1C). Study IV quantitatively assessed microbially-mediated nitrogen speciation and determined the abundance of functional genes involved.

**Fig. 1:**
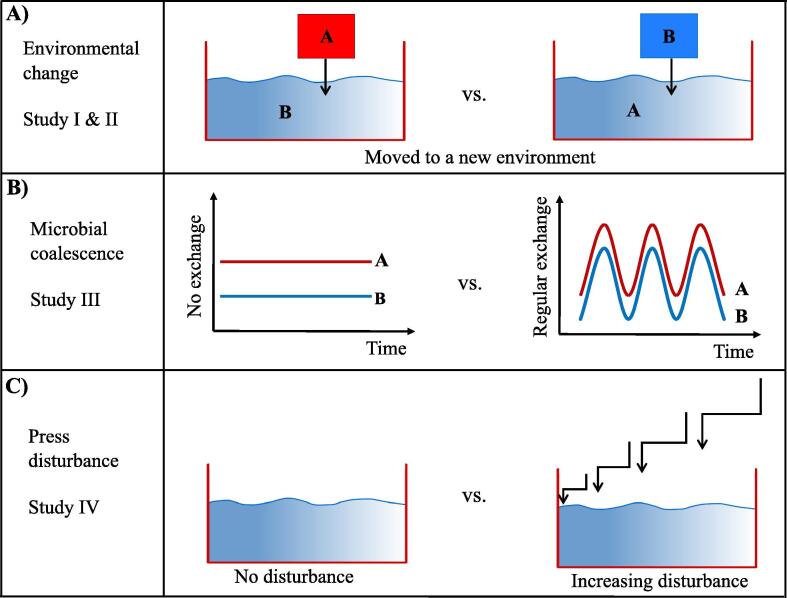
Scenarios of saltwater intrusion outlined in the four studies examining community assembly processes and functional outcome. A) community A and B are reciprocally transplanted between their environments using dialysis bags, which prevent the movement of microorganisms while subjecting the water to the abiotic conditions of the surroundings (such as salinity and nutrients). Hence, the two communities encounter a change in environmental conditions. B) In one setting, community A and B experience environmental change in a similar setup mentioned above, e.g., no exchange of microbial cells between the two communities. In another setting, cells of community A and B are exchanged regularly over a short period of time. The upside of this approach is that it considers presence of microbial coalescence in a close-to natural setting. C) Saltwater intrusion, classified as a press disturbance, is simulated in the setting of salt pulse over a period of two months. The varying levels of salt addition are carried out in the disturbance treatments, while the microcosms without salt addition serve as the control where a microbial community experiences no disturbance. The size of the arrows indicates the degree of salt pulse.

## 1 Study I: Community assembly mechanisms

In a laboratory study, I reciprocally exposed bacterioplankton from multiple salinity regions of the Baltic Sea, placing bacterioplankton from one environment in the environments hosting the others, thus exposing different communities to different salinities. This reciprocal transplant design enabled the impact of community composition on ecosystem functions to be distinguished from that of environmental conditions. In lieu of studying the individual responses of the thousands of species comprising bacterioplankton, I grouped the different taxa based on their response to disturbances and then identified the main assembly mechanisms for each group. A key prediction was that different assembly processes would be important for the dynamics of ecologically dissimilar taxa.

**Fig. 2:**
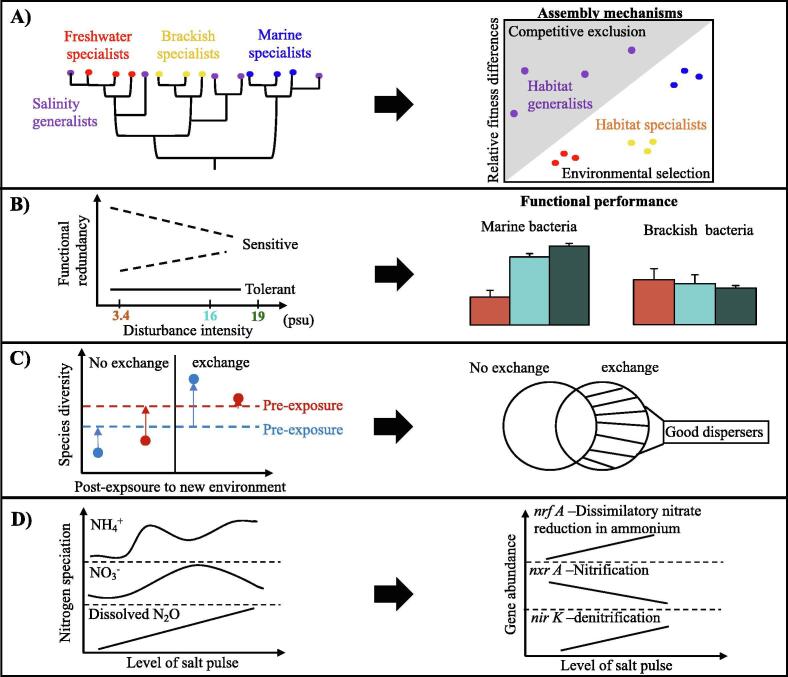
Outcome of community assembly and function. (A) The degrees of phylogenetic relatedness can be used to infer the ecological processes of community assembly. Accordingly, a higher phylogenetic relatedness among the taxa comprising each type of habitat specialist indicates that the strongest influence on their community assembly is exerted by environmental selection. ﻿Conversely, a lower phylogenetic relatedness of the species comprising habitat generalists indicates the greater relevance of competitive exclusion in generalist assembly, as it facilitates interactions between distantly related species. (B) Three possible scenarios for the relationship along disturbance intensity (the difference in salinity between each environment pair) and functional redundancy (FR): a community that tolerates changes in the surrounding environment expresses similar degrees of FR in response to increasing disturbance intensity. Conversely, a community sensitive to environmental changes expresses a higher or lower FR in response to greater disturbance intensity. Each bar with standard deviation on the right panel represents the FR of each community measured at a level of disturbance intensity. The outcome of FR differs between marine and brackish water bacteria. (C) Differently composed communities are indicated by red and blue circles, correspondingly. After exposure to a new environment, both of the non-exchanged communities have relatively low species diversity in comparison to the respective ones at pre-exposure indicated by dashed lines. However, a regular exchange results in a highest diversity of individual communities, compensating the loss of diversity that is caused by exposure *per se*. Good dispersers, present only in the exchanged communities (the unique fraction of the filled venn diagram), contribute to the increased diversity. (D) Changes in nitrogen (N) speciation and N-cycle related genes following salt pulses over a long period of time. (For interpretation of the references to colour in this figure legend, the reader is referred to the web version of this article.)

To investigate the underlying mechanisms, I made use of the phylogenetic approaches of macro-ecology to characterize community dynamics using DNA sequencing. Differences in the degree of phylogenetic relatedness reflect differences in relative fitness along environmental gradients [12]. The results showed that the freshwater, brackish and marine specialists were phylogenetically more closely related within a group than between groups, whereas generalists within a group were distantly related [6]. This finding suggested that environmental filtering strongly influences the assembly of habitat specialists, while competitive exclusion is more relevant for habitat generalists (Fig. 2A). In addition to identifying the assembly mechanisms of different community members, I found that 50% of the taxa within a community exhibited high abundance in native environment. The other half of the community members, most of which were present at low abundance in the native environment, were able to thrive after exposure to new salinity conditions.

## 2 Study II: The assessment of functional redundancy as community performance

Functional redundancy (FR) occurs when alterations in a community do not result in different process rates for a given ecosystem function [5]. It serves as an ecosystem buffer of functional performance for biological communities subjected to an environmental disturbance. To determine the extent that shifts in community composition affect ecosystem functions, coworkers and I analyzed the FR within disturbed communities from the above-described transplant experiment.

Using RNA sequencing and mathematical models, we quantified the changes in the abundances of individual genes expressed by each community member in pairwise comparisons of different environments and then calculated the degree of FR for each pair [7]. The results showed that the FR of marine bacteria was higher in response to greater environmental differences, whereas the FR of brackish water bacteria was consistent for any pairwise comparisons of the environments (Fig. 2B). This suggested that indigenous brackish bacteria better tolerate changes in salinity. Moreover, when all genes were grouped into broad and narrow functional categories, the FR was higher in the former. Together, these findings demonstrated the influence of the disturbance intensity on the FR of the disturbed communities, with a greater FR tending to compensate for the presence of more sensitive community members.

## 3 Study III: The contribution of dispersal to community outcome

In natural aquatic systems, microorganisms encounter constant water movement and competition with immigrants introduced by e.g., wind and ocean currents. To mimic these processes, I extended the experimental setup by comparing mixtures of bacterial cells from different environments of the St. Lawrence estuary with non-mixed bacteria and then examined the impact of exposure to new salinity environments on the bacterial community as well as the contribution of dispersal to community assembly [8]. I found that, even if exposure to the new salinity had caused a loss of diversity, the new level of diversity was maintained or even increased by dispersal, with the extent depending on the initial diversity and the metabolic plasticity of community members (Fig. 2C). Furthermore, I identified bacterial taxa with good dispersal capabilities based on presence-absence of taxa between non-exchanged and exchanged communities (Fig. 2C). These results suggest that dispersal enables an adjustment of taxonomic composition in the bacterial communities so that good dispersers are able to thrive and colonize the empty niches after exposure to salinity disturbance. My findings have important implications for microbial- engineering-based management, such as the maintenance of specific microbial communities in wastewater treatment plants and in biofilm development, where microbial biodiversity and colonization success during the short water-retention period are critical for an optimal system performance.

## 4 Study IV: The quantification of functional genes

The three studies mentioned above addressed how pulse disturbances (i.e., disturbances occur in a single event and over a short period of time) impacted community outcome, as bacterial communities experienced changes in salinity once. However, study IV investigated the role of a press disturbance (i.e., disturbances occur in multiple events and impact multiple generations of community members) influencing nitrogen transformation [9]. This assessment was critically important given Minjiang River closely links to drinking water availability in many coastal regions, particularly under climate change scenarios. In laboratory microcosms, my coworkers and I simulated salt addition of varying strength as a type of press disturbance to mimic saltwater intrusion to the river and quantified absolute abundance of functional genes involved in nitrogen cycles. The high saline environments led to a decrease in the concentrations of NO_3_^−^, and that reduction was mainly resulted from a decline of nitrification process and increased dissimilatory nitrate reduction to ammonium (DNRA) (Fig. 2D). As a result, NH_4_^+^ concentrations rose with increasing salt levels, and that was also supported by the enrichment of *nrf A* gene involved in DNRA following salt pulses (Fig. 2D). Our microcosms further demonstrate that denitrification can occur in oxic environments, and that increased salinity promoted the accumulation of dissolved N_2_O in the water.

## 5 Concluding remarks

**Fig. 3:**
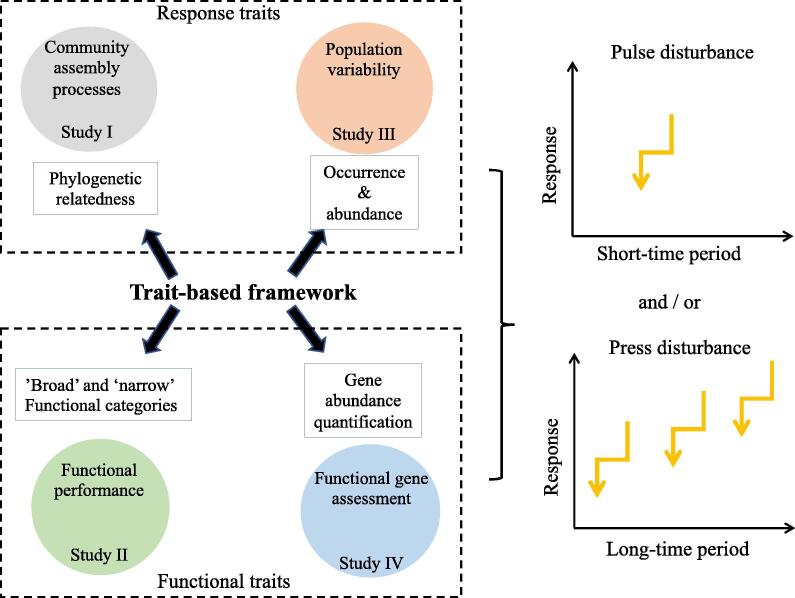
Schematic diagram illustrating the trait-based framework applied in the four studies for evaulation of microbial responses to pulse or press disturbance.

A common framework applied by the four studies was trait-based approaches, which is divided into two types: response and functional traits. Response traits of a species determine its abundance changes in a new environment; likewise, a community’s response traits can be an expression of changes in community composition. Study I and III assigned bacterial taxa into ecological groups whose response to disturbances was already documented when analyzing phylogenetic relatedness and occurence / relative abundance of the taxa, respectively (Fig. 3). In contrast, functional traits are species features that influence ecosystem properties or processes such as biogeochemical cycling [13]. Study II and IV measured functional traits by analyzing changes in abundances of transcripts or genes (Fig. 3). I believe, a trait-based framework exemplifies a useful ecological tool that enables researchers to decipher the complexity of the processes contributing to microbial community assembly and explore possible links between compositional and functional changes driven by pulse or press disturbances.

Collectively, the findings of the four studies shed light on the ecology and evolution of microorganisms in marine-freshwater transitions: bacteria can cross marine-freshwater boundaries, maintaining their taxonomic identity and functional outcome in response to the new environmental conditions to some extent. However, the findings cannot be rigorously extrapolated to natural environments, given that microbial microcosms have limited potentials to mirror the highly complex structural and temporal context of natural communities or for populations from different domains of life. Nonetheless, the findings can be interpreted as a general hint that disturbance intensity determines how a microbial community responds to environmental change, and that responses may be due to resource competition, innate resistance to disturbances and species interactions. I believe that this collection of studies will stimulate mechanistic hypotheses in microbial ecology that allow testing the robustness and functional stability of microbial communities under shifting environmental conditions in aquatic as well as other (eco)-systems [14].

## Funding

This research received no external funding.

## References

[1] Falkowski P.G., Fenchel T., Delong E.F. (2008). The microbial engines that drive Earth’s biogeochemical cycles. Science.

[2] Martiny J.B.H., Bohannan B.J.M., Brown J.H., Colwell R.K., Fuhrman J.A., Green J.L. (2006). Microbial biogeography: putting microorganisms on the map. Nat Rev Microbiol.

[3] Nemergut D.R., Schmidt S.K., Fukami T., O'Neill S.P., Bilinski T.M., Stanish L.F. (2013). Patterns and processes of microbial community assembly. Microbiol Mol Biol Rev.

[4] Hanson C.A., Fuhrman J.A., Horner-Devine M.C., Martiny J.B.H. (2012). Beyond biogeographic patterns: processes shaping the microbial landscape. Nat Rev Microbiol.

[5] Allison S.D., Martiny J.B.H. (2008). Resistance, resilience, and redundancy in microbial communities. Proc Natl Acad Sci U S A.

[6] Shen D., Jürgens K., Beier S. (2018). Experimental insights into the importance of ecologically dissimilar bacteria to community assembly along a salinity gradient. Enviro Microbiol.

[7] Beier S., Shen D., Schott T., Jürgens K. (2017). Metatranscriptomic data reveal the effect of different community properties on multifunctional redundancy. Mol Ecol.

[8] Shen D., Langenheder S., Jürgens K. (2018). Dispersal modifies the diversity and composition of active bacterial communities in response to a salinity disturbance. Front Microbiol.

[9] Xie R., Rao P., Pang Y., Shi C., Li J., Shen D. (2020). Salt intrusion alters nitrogen cycling in tidal reaches as determined in field and laboratory investigations. Sci Total Environ.

[10] Lozupone C.A., Knight R. (2007). Global patterns in bacterial diversity. Proc Natl Acad Sci U S A.

[11] Herbert E.R., Boon P., Burgin A.J., Neubauer S.C., Franklin R.B., Ardón M. (2015). A global perspective on wetland salinization: ecological consequences of a growing threat to freshwater wetlands. Ecosphere.

[12] Webb C.O., Ackerly D.D., McPeek M.A., Donoghue M.J. (2002). Phylogenies and community ecology. Annu Rev Ecol Syst.

[13] Srivastava D.S., Cadotte M.W., MacDonald A.A.M., Marushia R.G., Mirotchnick N. (2012). Phylogenetic diversity and the functioning of ecosystems. Ecol Lett.

[14] Cavicchioli R., Ripple W.J., Timmis K.N., Azam F., Bakken L., Baylis M. (2019). Scientists’ warning to humanity: microorganisms and climate change. Nat Rev Microbiol.

